# Size of cancer clinical trials and stopping rules.

**DOI:** 10.1038/bjc.1978.284

**Published:** 1978-12

**Authors:** S. J. Pocock

## Abstract

A recent international survey on the size of clinical trials in cancer showed the frequent problem of slow patient accrual, which remains a major hindrance to progress. The survey also revealed that, although the design of most trials specified a fixed number of patients, subsequent experience revealed a much more flexible approach, with analysis of results, say, every 4--6 months. Conventional sequential methods are hardly ever used and unfortunately most trials proceed without any predetermined stopping rules. Some trial organizers use repeated significance tests on accumulating data as a guide to the detection of treatment differences, an approach that can be adapted to a more rigorous statistical framework as a "group sequential design". The major statistical principle involved is that the more often one analyses the data the greater is the probability of achieving a statistically significant result, even when the two treatments are equally effective. Group sequential designs require the adoption of a more stringent significance level to allow for repeated testing. If one intends up to 10 repeated analyses of the data, only a treatment difference significant at the 1% level would merit a decision to stop the trial. For any trial to implement a stopping rule successfully there must also be prompt feedback and processing of response and survival data ready for up-to-date analysis. Such efficiency is often lacking. The repeated presentation of interim results of a trial to participating investigators can seriously affect their future reaction, especially if there are interesting but non-significant differences. Thus, some secrecy about ongoing results is advisable if trials are to achieve an unbiased conclusion.


*Br. J. Cancer* (1978) **38**, 757

### SIZE OF CANCER CLINICAL TRIALS AND STOPPING RULES

S. J. POCOCK

*From the Department of Clinical Epidemiology, Royal Free Hospital, Pond Street, London NW3*

Received 15 August 1978 Accepted 8 September 1978

**Summary.—A recent international survey on the size of clinical trials in cancer showed the frequent problem of slow patient accrual, which remains a major hindrance to progress.**

**The survey also revealed that, although the design of most trials specified a fixed number of patients, subsequent experience revealed a much more flexible approach, with analysis of results, say, every 4–6 months. Conventional sequential methods are hardly ever used and unfortunately most trials proceed without any predetermined stopping rules.**

**Some trial organizers use repeated significance tests on accumulating data as a guide to the detection of treatment differences, an approach that can be adapted to a more rigorous statistical framework as a "group sequential design". The major statistical principle involved is that the more often one analyses the data the greater is the probability of achieving a statistically significant result, even when the two treatments are equally effective.**

**Group sequential designs require the adoption of a more stringent significance level to allow for repeated testing. If one intends up to 10 repeated analyses of the data, only a treatment difference significant at the 1% level would merit a decision to stop the trial.**

**For any trial to implement a stopping rule successfully there must also be prompt feedback and processing of response and survival data ready for up-to-date analysis. Such efficiency is often lacking.**

**The repeated presentation of interim results of a trial to participating investigators can seriously affect their future reaction, especially if there are interesting but non-significant differences. Thus, some secrecy about ongoing results is advisable if trials are to achieve an unbiased conclusion.**

In the execution of any clinical trial for the treatment of cancer it is generally considered unethical and also very inefficient to wait until the results on all patients have been obtained before making any inferences about the effectiveness of treatments. In statistical parlance the "fixed sample size" approach is unacceptable. Nevertheless many trial protocols specify a fixed number of patients, often with certain statistical design objectives in mind, but one assumes from experience that the trial organizers do not intend to be as inflexible as they imply.

At the other extreme there exist methods of sequential design and analysis, as described in Armitage (1975), which are geared to the idea that after every additional patient on each treatment has been evaluated, some formal statistical stopping rules are applied to determine whether the whole trial should stop. Such a sequential approach is likely to involve certain assumptions:

(1) the response variable conforms to some standard statistical distribution,

(2) patients enter in matched pairs (one to each treatment);

(3) patient evaluation is instantaneous;

(4) constant surveillance is made of the accumulating data.

The situation for a typical cancer clinical trial is rather different:

(1) response is measured either by tumour shrinkage, disease-free interval or patient survival, any of which may have awkward distributions;

(2) patients vary with respect to several prognostic factors, and although stratification can be a partial solution, pairing of patients is impractical;

(3) responses such as those mentioned in (1) may take months or years to observe; moreover, patient entry from several hospitals will require a complex system of central data collection which entails some delay between patient evaluation at the hospital and inclusion of his outcome in the analysis;

(4) the statistician and trial organizers will normally be too busy to maintain a constant vigil over the data in order to observe the point at which a sequential boundary is crossed.

Hence there is a sharp contrast between the theoretical ideal of sequential methods and the practical situation of cancer clinical trials. This is certainly my own experience, since I am unaware of any cancer clinical trial which has successfully implemented a formal sequential design.

Therefore neither fixed sample size nor conventional sequential methods are applicable. Instead the cancer clinical trial will normally proceed with some inspection of results from time to time with an informal interpretation by the trial organizers as to whether any action to stop or change a treatment is justified. Some fixed sample-size significance tests may be used to aid decision making, but the overall approach is subjective. One might take the defeatist standpoint that *any* more formalized approach to the design and analysis of trials is unrealistic. However, I think this is liable to result in a very unscientific approach to clinical trials, whereby the statistical validity of conclusions would remain uncertain.

What is needed is a method of statistical design and analysis which takes into account the fact that it is common practice to assess the accumulating results of an ongoing trial at several equally spaced times, each of which might normally occur before a meeting of the trial organizers. This paper describes one such general type of group sequential design based on the repeated use of conventional significance testing.

However, before we go into the details of such an approach, we should consider the current status of clinical trial practice.

### A SURVEY ON THE SIZE OF CANCER TRIALS

This survey was undertaken for the UICC Project on Controlled Therapeutic Trials by Pocock *et al.* (1978). The main questions of the survey were:

(1) Do investigators determine the required size of trial in advance?

(2) How successful are they in accruing an adequate number of patients?

(3) Do they assess interim results while the trial is in progress, and do such analyses affect the eventual size of trial?

The sampling frame for the survey was the 334 trials registered with the UICC information office from 1972 to mid-1975. A random sample of 50 trials was chosen, and a questionnaire sent to each principal investigator. Completed questionnaires were received from 40 (80%). Eighteen of these were in Western Europe, 17 in the United States and 5 elsewhere. Twenty-eight of the trials accrued patients from more than one centre, and 17 of these had more than 20 participating centres. All trials were randomized, this being a condition for inclusion in the UICC register. Twenty-eight of the trials had just 2 randomized treatment groups. Twenty-six trials were for chemotherapy, which is probably a true reflection of current trial activity. The trials covered a wide range of tumour sites, including 9 for leukaemia.

The replies to the most relevant questions are presented in 3 sections:

### (*a*) *Design*

In 34/40 cases (85%) the required number of patients was specified before the trial began. The actual number was recorded in 26 cases and the distribution was as follows:

| | | |
|---|---|---|
| 9 trials required | 45–90 patients | (all |
| 12 | 100–200 | treatments |
| 1 | 210 | combined) |
| 1 | 400+ | |
| 2 | 500 | |
| 1 | 1690 | |

The methods of determining the required sample size were as follows:

18 used power calculations for a fixed number of patients;
8 made a subjective decision, without statistical methods;
1 used a sequential design, which was abandoned later;
3 made somewhat unusual statistical statements; and
4 used statistical methods, with no details given.

Thus, statistical power calculations for determining a fixed size of trial seems the most popular approach. This involves:

(1) making a decision as to what is the smallest difference in treatment effects which it is important to detect;

(2) defining a single significance test and level (say $P < 0{\cdot}05$) to be used once only at the end of the trial as the criterion for detecting that a difference exists; and

(3) specifying a degree of certainty (say 90%) that the detection method (2) for the underlying difference (1) would be successful. The required number of patients can thence be obtained from a simple statistical formula.

This method can give rise to a very wide range of sample size needs, as illustrated in this survey. The 1690 patients required in one trial reflects a statistician's excessive adherence to this approach, without consideration of what was a feasible rate of accruing patients. A more realistic approach is normally adopted, whereby the power calculations are made to fit a preliminary subjective decision on how long a trial should last and the availability of suitable patients. Thus, the statistical methods are used as a check on the scientific acceptability of a choice already made on practical grounds. This seems reasonable provided the statistical statements (1)–(3) above do not become too optimistic. Unfortunately, in 8 of the above trials a 100% difference in median survival or median disease-free interval was used as the basis for power calculations, which resulted in relatively small sample-size requirements of around 30–50 patients per treatment. This enthusiasm of investigators for small Phase III trials in the hope of very large treatment differences is a considerable hindrance to progress in cancer research.

(*b*) *Realization*

At the time of this survey only half of the trials had terminated patient accrual, so that one cannot give an overall picture of their eventual outcome. However, since all trials had been in progress for at least 2 years one can study the mean annual accrual rates. This information was available for 39 trials, and the distribution is as follows:

| | |
|---|---|
| 3 trials entered | < 10 patients/annum |
| 8 | 10–19 |
| 6 | 20–29 |
| 8 | 30–49 |
| 8 | 50–79 |
| 5 | 100–199 |
| 1 | 266 |

Another way of considering a trial's progress is to calculate the number of years of patient accrual required to achieve the target number specified in the trial design. This information was available for 24 trials and the distribution is as follows:

| | |
|---|---|
| 3 trials require | under 2 years |
| 2 | 2–3 years |
| 7 | 3–4 years |
| 7 | 4–8 years |
| 5 | 10 years. |

In summary, the median accrual rate is 33 patients per annum and the median time to achieve the specified accrual target is over 4 years. Clearly, this is a very unsatisfactory situation, which results in many trials either failing to achieve adequate patient numbers or being excessively protracted. Further study of the survey results indicates that both single-institution and multicentre trials experience these problems. Until all trial organizers obtain a truly realistic assessment of the potential patient accrual, and ensure the full cooperation of all contributing investigators, the problem of poor accrual will continue to ruin a large proportion of clinical trials.

(*c*) *Analysis*

Investigators were asked whether they had undertaken any form of *interim or ongoing analysis* of results while the trial was in progress. 33/40 (83%) responded Yes and the frequency of interim analysis was as follows:

| | |
|---|---|
| Every 3 months | 1 |
| Every 4 months | 9 |
| Every 6 months | 13 |
| Every year | 3 |
| Every 2 years or less | 2 |
| Sequential analysis | 2 |
| One toxicity analysis only | 2 |
| Unknown | 1 |

Evaluation of the trial every 4–6 months seems a very common practice, which is probably linked to the tri- or bi-annual meetings of the trial organizers. One of the two sequential analyses was eventually abandoned, whilst the other involved a separate sequential treatment comparison within each of several patient strata with reporting of results every 3 months, so that neither remained truly sequential. Six of the 7 who had not undertaken interim analyses implied they would do so once there was sufficient data.

Investigators were also asked whether they used any formal or informal *stopping rules* for the early termination of trial if treatment differences should develop. The 38 replies were as follows:

22 had no stopping rules
2 used sequential methods, as mentioned earlier
6 used repeated significance testing
4 adopted a subjective approach based on the magnitude of treatment difference
1 used a peculiar statistical argument
3 used some stopping rule, but gave no details.

Thus the majority had no agreed policy as to early termination of trial. This is unfortunate since the whole object is to identify a superior treatment and to ensure that patients will not receive an inferior treatment once a difference is clearly established.

Repeated significance testing means that at periodic intervals, say every 6 months, one or more significance tests are carried out to see whether there is evidence of a treatment difference. If statistical significance is reached at some point, this will be used as the basis for a decision to stop the trial. This approach seems quite sensible in that, unlike many sequential designs, it is readily understood by both statisticians and clinicians. However, there are 3 major problems which need to be clarified:

(1) If there are several measures of a patient's response to treatment, *e.g.* tumour shrinkage, survival, toxicity, disease-free interval, then it is quite likely that some will show significant differences and others will not. This presents a logical problem which may be overcome by giving primary importance to one variable and using other comparisons as a more informal check that the superiority of one treatment follows a consistent pattern. Alternatively, one could define some single more complex multivariate significance test based on all relevant variables, but this may be unrealistic in the case of such disparate factors as tumour response and toxicity.

(2) One cannot expect clinical investigators to accept a significance test as the sole criterion for stopping a trial. Previous experience, evidence from trials in other centres and the degree of enthusiasm for the trial, will all necessarily be taken into account. Thus the ultimate decision will be a subjective one but it is important that statistical evidence be a primary factor.

(3) The more often one performs a significance test on the accumulating results in a trial, the greater is the chance that some significant difference will eventually be detected, even if the treatments are really equally effective. This fact will tend to contribute to an excess of false positive results reported in the clinical trial literature. Hence repeated examination of data means that one must set a more stringent significance level than $P < 0{\cdot}05$, and this point is discussed further in the next section. However, before we approach this more statistical topic it may be helpful to state the following simple rule:

If one expects to take up a maximum of 10 repeated looks at one's data during the course of a trial, then one might wish to adopt a significance level of $P < 0{\cdot}01$ as the criterion for stopping the trial, since the chances of drawing a *false* conclusion that one treatment is superior is roughly equivalent to making a decision based on a single test at the level $P < 0{\cdot}05$.

### GROUP SEQUENTIAL ANALYSIS

This section describes how repeated significance testing of accumulating data can be formulated as a precise method of statistical analysis for clinical trials. Armitage (1975) describes several RST sequential designs based on significance testing after each pair of patients, one to each treatment. However, as mentioned in Section 1, this continual testing has both theoretical and practical difficulties. Instead we consider the *group sequential* approach, whereby significance tests are performed at longer equally spaced intervals. The results and methods described here are based on Pocock (1977) and further reference to the same general approach can be obtained from McPherson (1974, 1977).

First, we return to the problem that repeated significance tests increase the *overall significance level*, that is, the probability of at least one significant difference when the treatments are really the same. Table I shows the results for

TABLE I.—*Repeated significance tests on accumulating data (two treatments and a normal response variable)*

| No. of repeated significance tests at the 5% level | Overall significance level |
|---|---|
| 1 | 0·05 |
| 2 | 0·08 |
| 3 | 0·11 |
| 4 | 0·13 |
| 5 | 0·14 |
| 10 | 0·19 |
| 20 | 0·25 |
| 50 | 0·32 |
| 100 | 0·37 |
| 1000 | 0·53 |
| ∞ | 1 |

repeated two-sided testing at the 5% level at equally spaced numbers of patients with two treatments, and a normally distributed response variable with constant known variance, though broadly similar results hold for any type of response variable and any pattern of repeated testing. For only 10 repeated tests the overall significance level has increased from 0·05 to 0·19, and with more and more repeated testing one can become increasingly sure of declaring a treatment difference whether one is really present or not. Clearly the naïve application of repeated significance testing allows the unscrupulous investigator ample scope to demonstrate some significant advance in the treatment of cancer!

The way to correct for this problem is to choose a more stringent *nominal significance level* for each repeated test, so that the overall significance level is 0·05. Table II shows what these nominal levels need to be for the case of a normal response, though simulation has shown that the same nominal levels are accurate

TABLE II.—*Nominal significance levels corresponding to an overall significance level of* 0·05 (*normal response*)

| No. of repeated significance tests | Nominal significance level |
|---|---|
| 2 | 0·0294 |
| 3 | 0·0221 |
| 4 | 0·0182 |
| 5 | 0·0158 |
| 10 | 0·0106 |
| 15 | 0·0086 |
| 20 | 0·0075 |

for a wide variety of response variables. Furthermore, these results hold for more elaborate tests involving adjustment for covariates, and are not sensitive to minor variations away from exactly equal numbers of additional patients between tests.

This simple adjustment can thus make repeated significance testing a respectable statistical tool, the only restriction being that one must decide in advance how many repeated tests are to be performed.

Let us illustrate the approach with data from an actual trial of two drug combinations, CP (cytoxan–prednisone) and CVP (cytoxan–vincristine–prednisone), for the treatment of advanced non-Hodgkin's lymphoma. The main criterion for response was tumour shrinkage, and the Figure shows how the response rates on the two treatments varied over the course of the trial, patient accrual being from June 1972 to October 1974. It can be seen that every time a patient is evaluated the response rate changes and, as in the early stages of any trial, this leads to wild fluctuations. However, as patient numbers increase, the curves inevitably become more stable.

Now, suppose the intention was to have around 120–130 patients in the trial and to analyse the accumulating data on 5 occasions, *i.e.* after about every 25 patients. This would lead to analyses at the times marked ↑ in the Figure with the following results:

FIG.—Cumulative response rates for treatments in a clinical trial for advanced non-Hodgkin's lymphoma.

| | Response rates CP | CVP | $\chi^2$ (without continuity correction) | $P$ |
|---|---|---|---|---|
| January 1973 | 3/14 | 5/11 | 1·63 | |
| July 1973 | 11/27 | 13/24 | 0·92 | |
| November 1973 | 18/40 | 17/36 | 0·04 | |
| April 1974 | 18/54 | 24/48 | 3·25 | >0·05 <0·1 |
| October 1974 | 23/67 | 31/59 | 4·25 | >0·0158 <0·05 |

At each of the 5 times the response rates are compared using a $\chi^2$ test without continuity correction, with the intention of stopping the trial if $P < 0{\cdot}0158$, the nominal significance level obtained from Table II. The lack of continuity correction is necessary to avoid the repeated testing being unduly conservative (see Pocock, 1977). Even with $P < 0{\cdot}05$ on the final test, one could not declare a treatment difference significant at the 5% level, since the nominal $P$ value of 0·0158 was not achieved. Of course, one would not wish to take a totally negative interpretation for this trial. In practice one would infer from this data alone that the superiority of CVP with regard to tumour response is interesting but inconclusive. Eventually, further data on the duration of response and patient survival would help to clarify the situation.

Superficially, this example illustrates the ease with which group sequential methods can be used. In fact, there are several difficulties that need to be raised. Tumour response is not seen instantaneously, and can take up to several weeks to be seen. The simplest solution is to allow a fixed period, say 3 months, to observe whether response occurs in a patient. This means that analysis after each group of patients takes place 3 months later than was indicated above, *i.e.* the first analysis would have been made in April 1973, not January 1973. Such an unavoidable delay means that further patients will have entered the trial in the interim, and this may raise complications if the nominal significance level is achieved and the trial is stopped. If stopping the trial means that all patients still receiving the inferior treatment are taken off it instantaneously, there will be no further direct data on response and the treatment comparison remains unaltered. However, if it is thought appropriate for patients entered but not evaluated to complete their current therapy, there will be further response data which may slightly alter the final treatment comparison. This can lead to contradictions if the results become less significant, but should not be a serious problem unless the delay to observe response is unduly long.

In this regard, there may be administrative delay in getting the observed response reported for inclusion in analysis. In multicentre cooperative groups this can be a matter of months, in which case any stopping rule becomes greatly delayed. For instance, the above example illustrates how one would have liked to conduct the ongoing analysis of the trial, whereas in practice the delays were such that the final response data were not analysed until over a year after the last patient was entered. It seems to me that for the benefit of patients participating in clinical trials there must be a considerable improvement in the feedback and processing of response data, in order that a prompt analysis can be carried out and any inferior treatment detected.

### GROUP SEQUENTIAL DESIGNS

We now consider how the method of repeated significance testing can be formulated into the design of a clinical trial, particularly as regards power calculations. The two features to be decided on at the start of such a group sequential trial are:

(a) How many significance tests should there be, *i.e.* what is the maximum number of groups?

(b) How many patients should be evaluated before each significance test, *i.e.* what should be the size of each group?

Let us here consider a trial with two treatments, $2n$ patients per group ($n$ per treatment) and a maximum of $N$ groups. This makes the maximum size of trial $2nN$.

The method of determining the operating characteristics of designs with a variety of values for $n$ and $N$ is described by Pocock (1977). Here we consider the simplest theoretical case of two treatment groups, for each of which we have a normally distributed response with means $\mu_A$, $\mu_B$ and known variance $\sigma^2$. The conventional power calculation here requires specification of an overall significance level $\alpha$ and power $1-\beta$ for a specific alternative hypothesis $\mu_A - \mu_B = \delta$. Tables derived by numerical integration enable the required value of $n$ for any given $N$ to be determined, but for limitations of space let us here just consider results for $\alpha = 0{\cdot}05$ and $1-\beta = 0{\cdot}9$ presented in Table III. Remember that the required nominal significance levels for any choice of $N$ are to be found in Table II. Clearly, as the number of groups $N$ increases, the number per group $2n$ decreases and the maximum number of patients $2nN$ increases.

This means that the larger is $N$ for a given $\alpha$ and $\beta$, the longer the trial will take to complete if the null hypothesis of no treatment difference appears to be true. Table III shows that in this situation 20% more patients will be needed for a design with $N = 5$ compared to a "one-look" trial ($N = 1$).

However, this is compensated by the most important feature in a group sequential design, which is the extent to which it enables early termination of trial when the alternative hypothesis is true. This is indicated in the last column of Table III by the average sample size. Evidently the greatest reduction is achieved by using a 2-group design instead of a 1-group (i.e. fixed sample size) design and there appears little advantage in using a design with more than 5 groups. This applies to any trial design based on $\alpha = 0{\cdot}05$ and $1-\beta = 0{\cdot}9$, and similar examples could be evaluated for other values of $\alpha$ and $\beta$. The only advantage of designs with a large number of groups (*i.e.* repeated significance tests) is in the early detection of extremely large treatment differences. Consider one extreme case of repeated significance testing after every pair of patients (*i.e.* an RST sequential design) for $\alpha = 0{\cdot}05$, $1-\beta = 0{\cdot}9$ and $\delta = 0{\cdot}5\sigma$. The required maximum sample size is 242 and the average sample size under $H_A$ is 116·6 compared with 115·2 for the equivalent 5-group design. Thus, continual significance testing has no statistical advantage compared with occasional testing at 5 equally spaced intervals.

It has been suggested that the nominal significance level could be varied over the $N$ tests, possibly having more stringent tests early on in the trial, but it is unclear what statistical advantage this might have to compensate for the increased complexity of design.

Of course, the response variable in a

TABLE III—*Group sequential designs for a normal response with known variance $\sigma^2$, overall significance level $\alpha = 0{\cdot}05$ and power $1-\beta = 0{\cdot}9$ under $H_A$: $\mu_A - \mu_B = \delta$*

| Maximum No. of groups ($N$) | Required No. of patients per group ($2n$) | Maximum No. of patients ($2nN$) | Average No. of patients to termination of trial under $H_A$ |
|---|---|---|---|
| 1 | 42·04 | 42·04 | 42·04 |
| 2 | 23·12 | 46·24 | 32·60 |
| 3 | 16·11 | 48·33 | 30·29 |
| 4 | 12·43 | 49·72 | 29·33 |
| 5 | 10·14 | 50·70 | 28·80 |
| 10 | 5·35 | 53·50 | 28·03 |
| 20 | 2·79 | 55·80 | 27·98 |
| | $\times \frac{\sigma^2}{\delta^2}$ | $\times \frac{\sigma^2}{\delta^2}$ | $\times \frac{\sigma^2}{\delta^2}$ |

clinical trial rarely follows a normal distribution, but for trials with relatively few groups this is not a serious problem, since asymptotic normal approximations can be used with sufficient accuracy. In particular, Pocock (1977) describes how binary and exponential responses can be used for group sequential designs. Also, in most trials it is important to allow for prognostic factors in making treatment comparisons, but provided one does not include too many factors, the inclusion of covariate adjustment in analysis will have no serious effect on the operating characteristics of group sequential designs.

### SURVIVAL DATA

It is becoming widely accepted that most survival data are best analysed by non-parametric methods. Armitage (1975) and Jones & Whitehead (in press) have considered sequential analysis based on the log–rank test. However, repeated analysis after every death would be an exhausting exercise and instead I wish to consider here how a group sequential approach could be used.

The usual group sequential testing described in Sections 3 and 4 is at equally spaced numbers of patients, whereas for the log–rank test it would seem more appropriate, and asymptotically equivalent, to analyse at *equally spaced numbers of deaths*. The theoretical details have not been worked out yet but I believe that the nominal significance levels in Table II would provide an overall significance level of 0·05 for a log–rank group sequential design. Further research would also be needed to define power calculations in this context.

Such an approach would mean that a considerable time would elapse between the start of the trial and the first analysis, but the time intervals between analyses would then shorten as more patients were entered and deaths occured more frequently. In this way one would avoid any unduly premature survival analyses based on very few deaths.

The success of group sequential survival designs primarily depends on the speed with which deaths are reported. The consequences of an early significant result may just be cessation of patient entry, but if treatment is ongoing (*e.g.* long-term chemotherapy) use of the inferior treatment on patients already entered may also cease, so that the whole trial is closed. In this latter case, there will be no further data to add to the survival comparison, except as a result of administrative delay, but in the former case there will be greater difficulty of statistical interpretation as further survival follow-up continues. This problem of a stopping rule being followed by further data has not been satisfactorily resolved but the best approach may be a conventional fixed-sample-size analysis of the final data with some informal acknowledgement that a stopping rule has been used.

### SECRECY OF INTERIM ANALYSES

Repeated presentation of interim results can have considerable influence on the participating investigators. An early interim analysis showing treatment comparisons can have disastrous effects on the future progress of a trial. If there is little difference between treatments some investigators may lose interest. However, a more serious situation arises if there are interesting but non-significant differences. Some participants may then drop out of the trial arguing that they believe there is a genuine difference, while others may continue in a half-hearted manner with perhaps an increased tendency to adapt the supposedly inferior treatment, remove patients from it prematurely or, worse still, interfere with the randomization.

Investigators may not be happy with complete secrecy, so a compromise solution may be required whereby results are presented for all treatments, combined with an additional statement that there is no significant difference as yet. Some small committee perhaps made up of one statistician and a non-participating clinician could keep a detailed check on the interim results which would only be presented to others in full when treatment differences are of sufficient magnitude to merit termination of the trial.

An additional problem is the premature publication of results while a trial is still in progress, which has the more serious effect that the whole medical community may be prejudiced towards a particular conclusion before the full results are known. Such early public presentation took place in at least 7 of the 40 survey trials mentioned above.

### CONCLUSIONS

(1) For most clinical trials in cancer, some form of informal ongoing analysis of results is undertaken, though conventional sequential methods are hardly ever used.

(2) This practice of periodically analysing the accumulating data can be formulated more precisely as a group sequential design, whereby stopping rules are based on repeated significance testing at equal intervals. There appears no great advantage in carrying out many repeated tests, both for statistical reasons and because of the effort involved. One sensible design would be to plan for no more than 10 repeated analyses, with a decision to stop the trial if the main treatment difference is significant at the 1% level.

(3) Such statistical stopping rules can never be rigorously applied but should improve the objectivity of decision-making.

(4) Difficulties in obtaining adequate patient accrual, administrative inefficiency and premature dissemination of results are major faults in the organization of many cancer trials for which no amount of statistical refinement can correct.

I am grateful to Professor P. Armitage for helpful advice.

### REFERENCES

Armitage, P. (1975) *Sequential Medical Trials*. Oxford: Blackwell.

McPherson, K. (1974) Statistics: the problem of examining accumulating data more than once. *N. Engl. J. Med.*, **290**, 501.

McPherson, K. (1977) Sequential analysis of clinical trial data. In *Clinical Trials*, Chapter 6, Eds. F. N. and S. Johnson. Oxford: Blackwell.

Pocock, S. J. (1977) Group sequential methods in the design and analysis of clinical trials. *Biometrika*, **64**, 191.

Pocock, S. J., Armitage, P. & Galton, D. A. G. (1978) The size of cancer clinical trials: an international survey. *UICC Tech. Rep. Series*, **36**, 5.


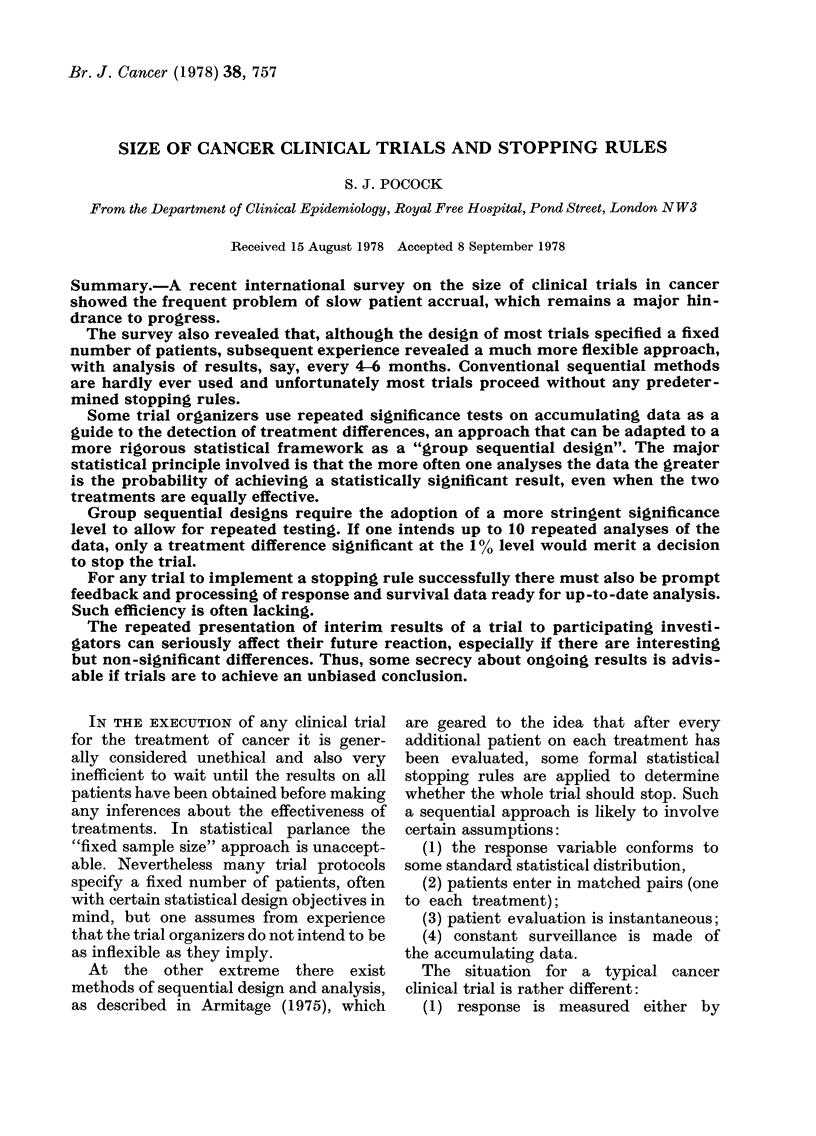

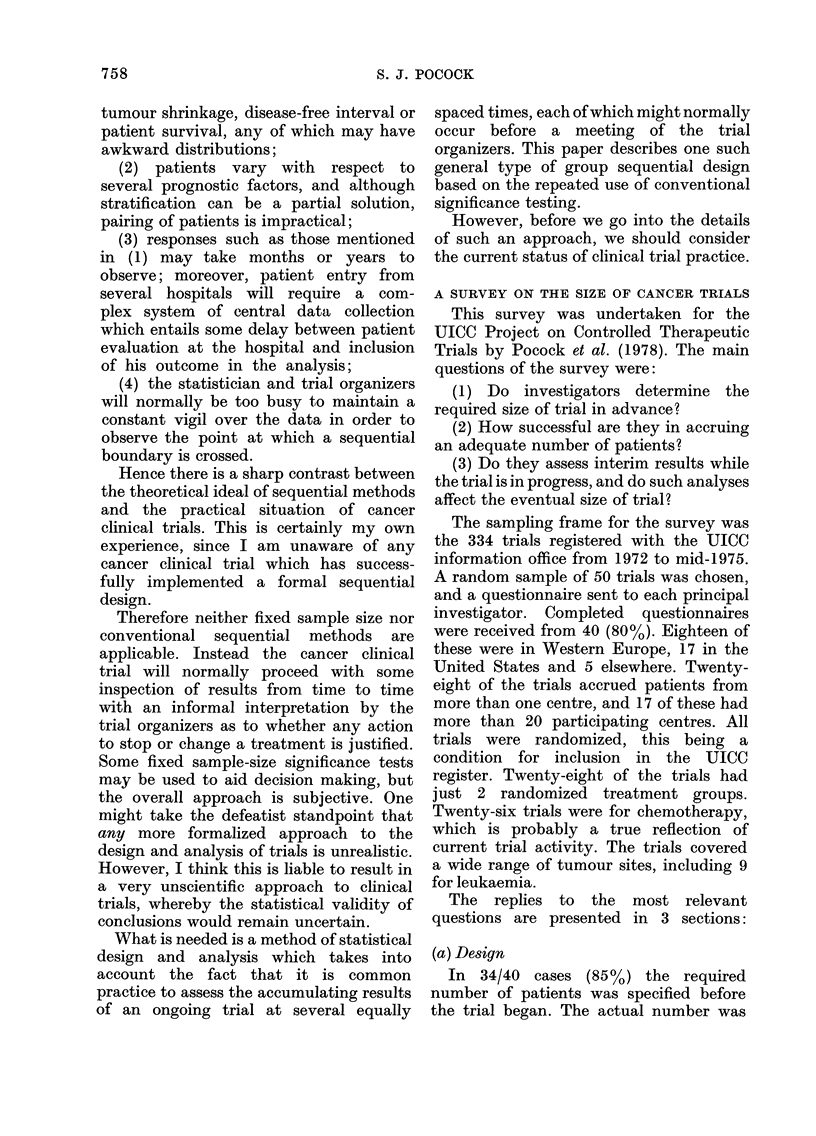

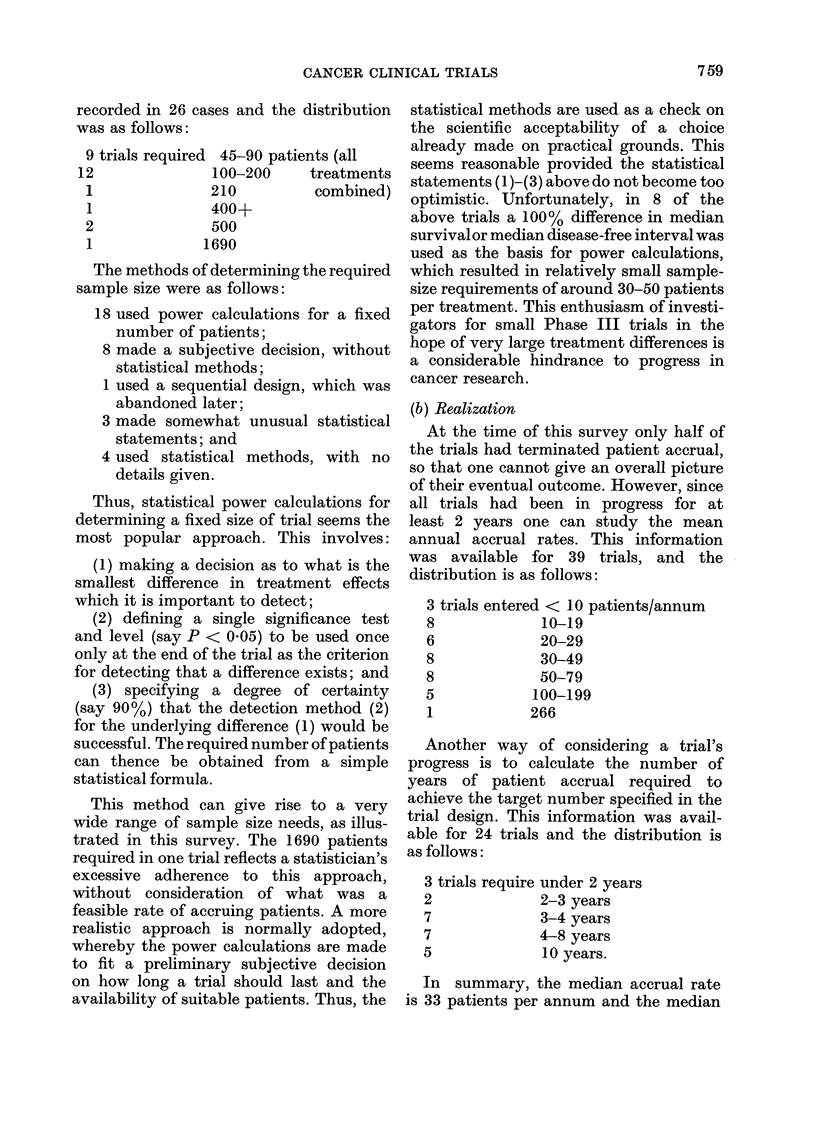

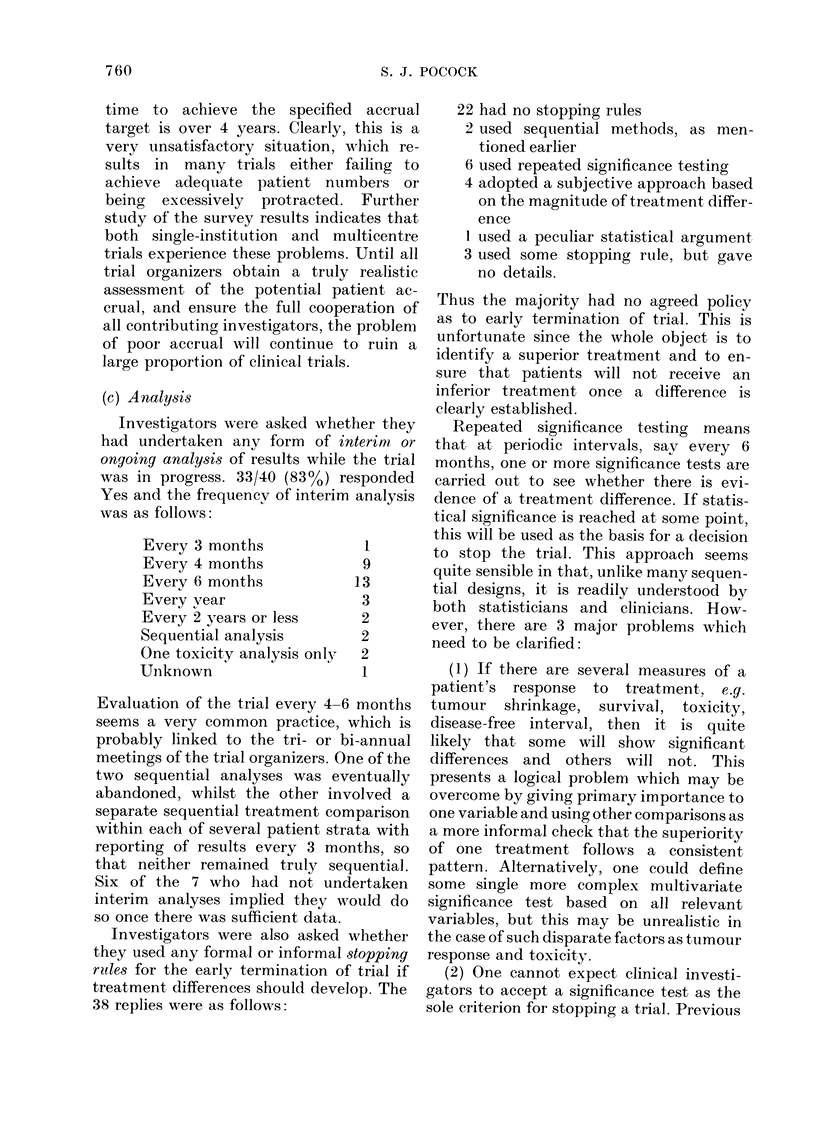

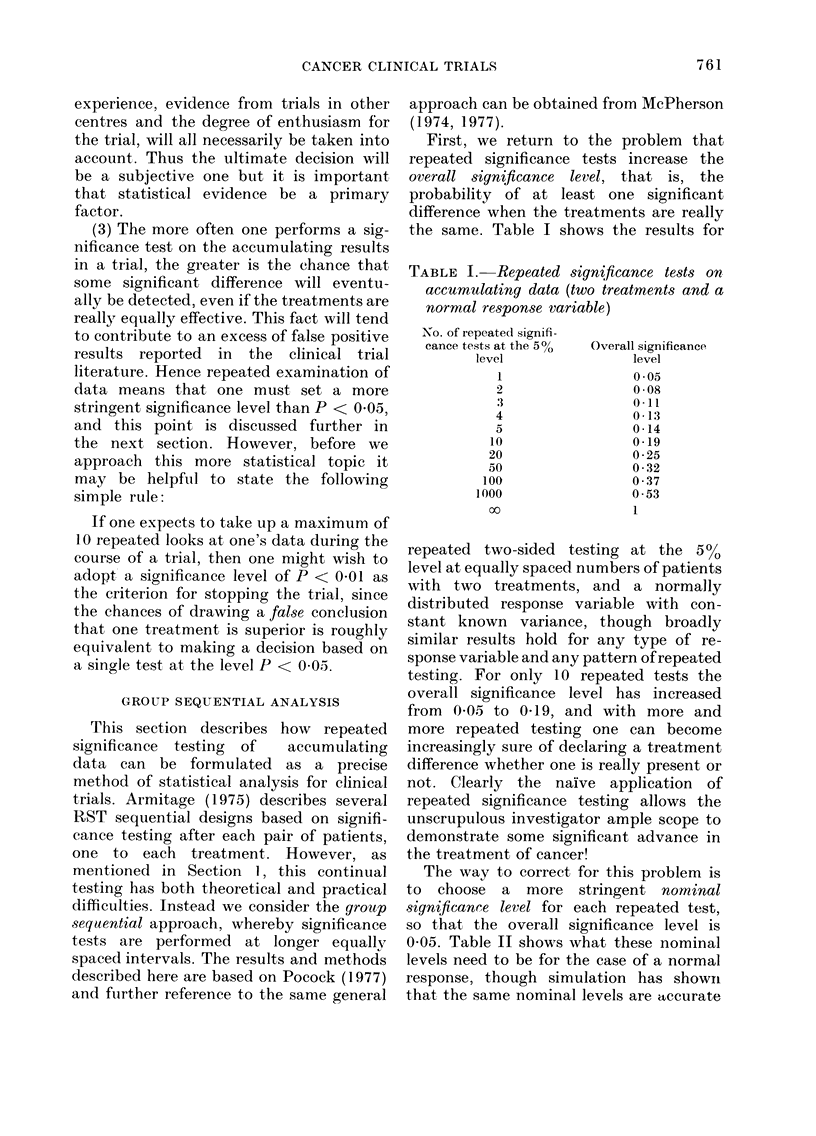

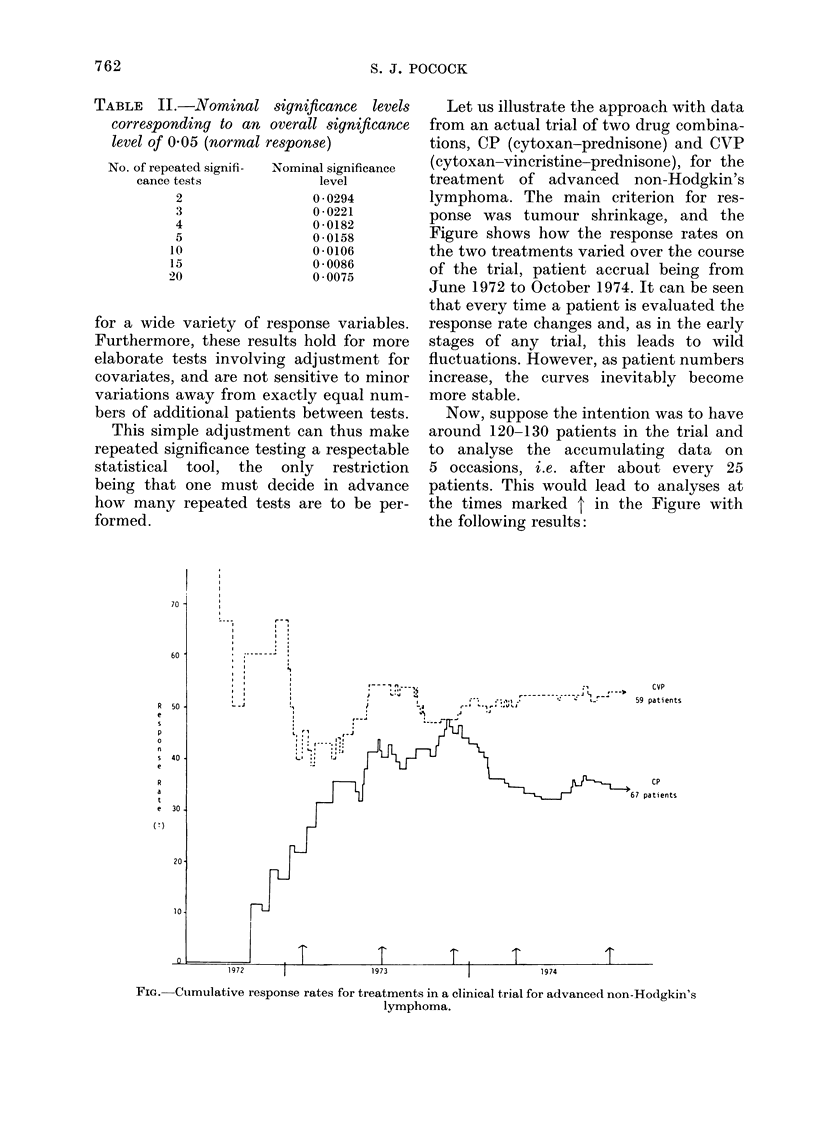

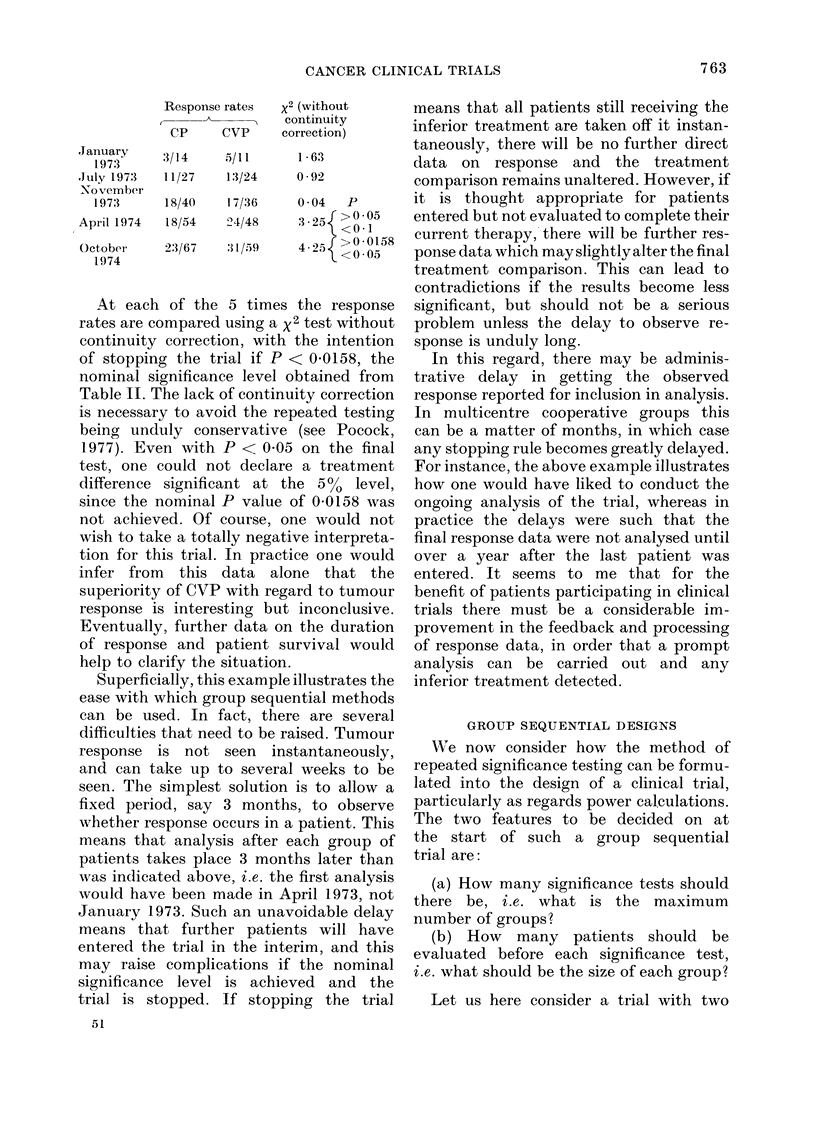

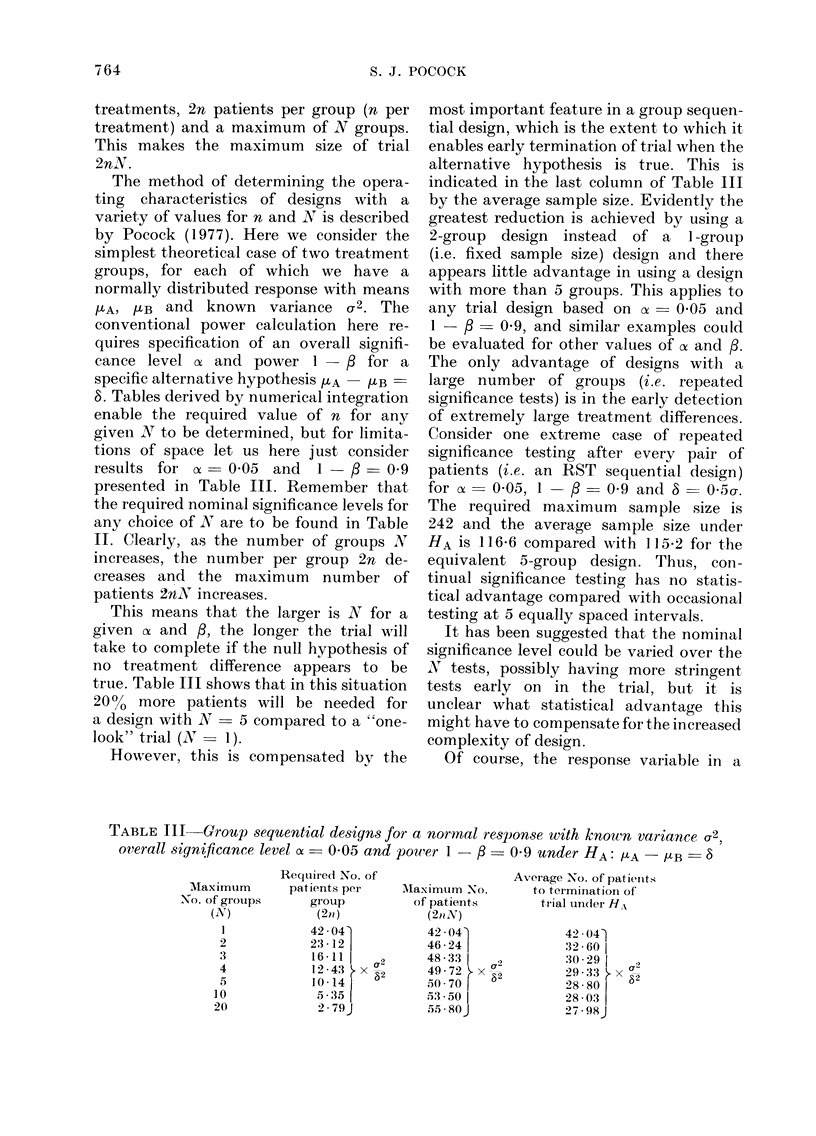

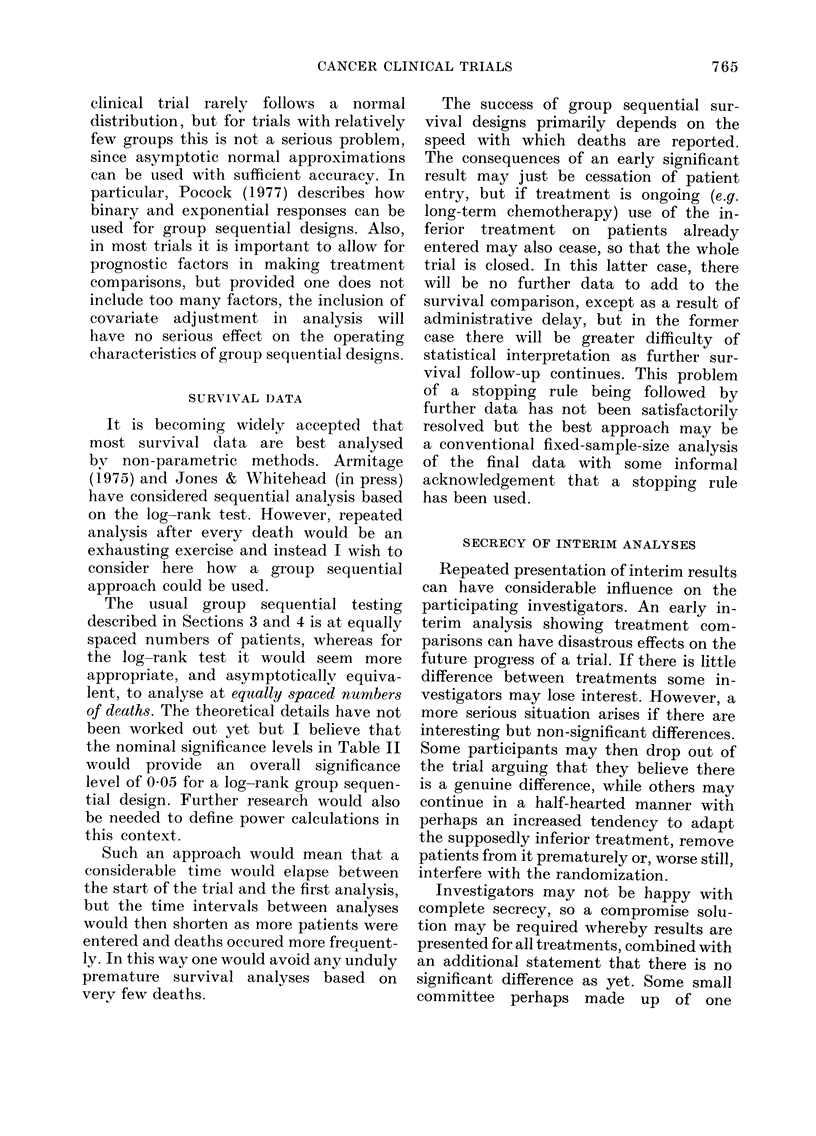

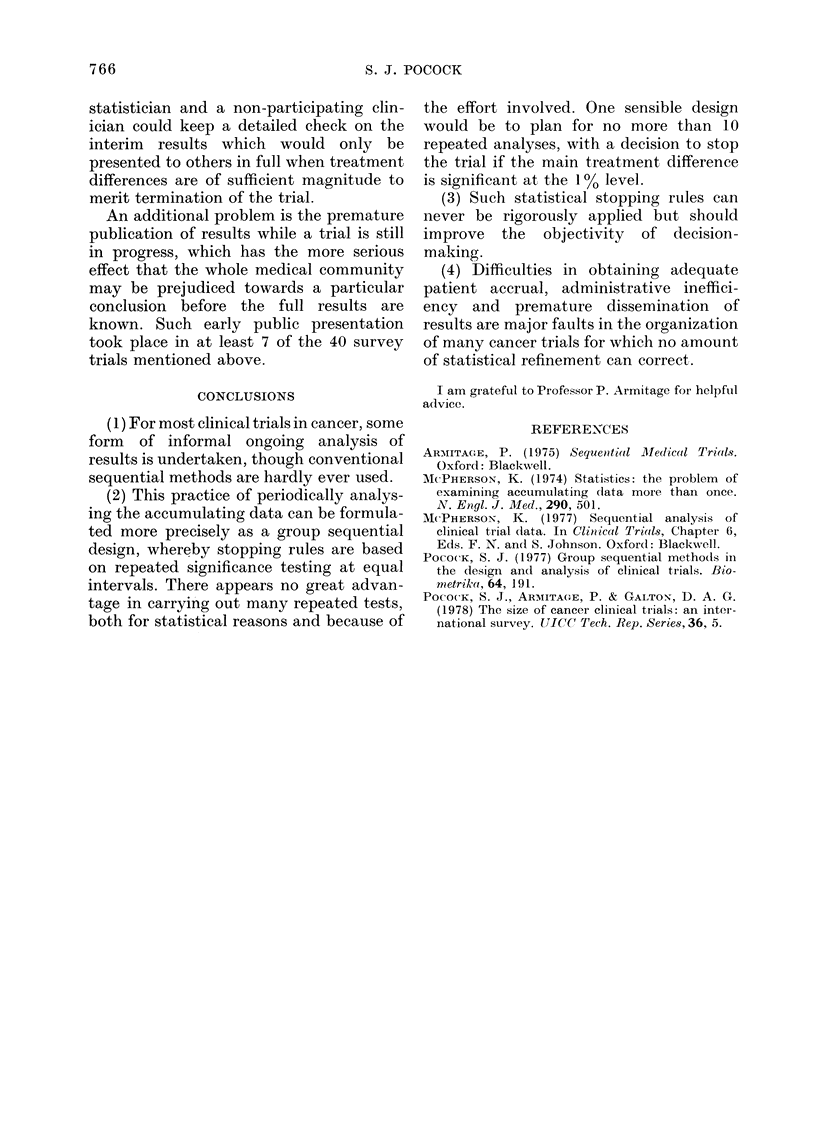


## References

[OCR_01174] McPherson K. (1974). Statistics: the problem of examining accumulating data more than once.. N Engl J Med.

